# Attributable Failure of First-line Cancer Treatment and Incremental Costs Associated With Smoking by Patients With Cancer

**DOI:** 10.1001/jamanetworkopen.2019.1703

**Published:** 2019-04-05

**Authors:** Graham W. Warren, Kathleen B. Cartmell, Elizabeth Garrett-Mayer, Ramzi G. Salloum, K. Michael Cummings

**Affiliations:** 1 Department of Radiation Oncology, Medical University of South Carolina, Charleston; 2 Department of Cell and Molecular Pharmacology, Medical University of South Carolina, Charleston; 3 Department of Nursing, Medical University of South Carolina, Charleston; 4 Department of Public Health Sciences, Medical University of South Carolina, Charleston; 5 Department of Health Outcomes and Biomedical Informatics, University of Florida, Gainesville; 6 Department of Psychiatry and Behavioral Sciences, Medical University of South Carolina, Charleston

## Abstract

**Importance:**

Previous studies have shown that continued smoking among patients with cancer can increase overall and cancer-specific mortality, risk for second primary cancer, and risk for toxic effects of cancer treatment. To our knowledge, there have been no efforts to estimate additional costs for cancer treatment attributed to smoking.

**Objective:**

To model attributable incremental costs of subsequent cancer treatment associated with continued smoking by patients with cancer.

**Design, Setting, and Participants:**

For this economic evaluation, a model was developed in 2018 using data from a 2014 US Surgeon General’s report that considered expected failure rates of first-line cancer treatment in nonsmoking patients, smoking prevalence, odds ratio of first-line cancer treatment failure attributed to smoking compared with nonsmoking, and cost of cancer treatment after failure of first-line cancer treatment.

**Main Outcomes and Measures:**

Attributable failures of first-line cancer treatment and incremental cost for subsequent treatment associated with continued smoking among patients with cancer.

**Results:**

Attributable treatment failures were higher under conditions in which high first-line cure rates were expected in nonsmoking patients compared with conditions in which low cure rates were expected. Peak attributable failures occurred under the conditions in which expected cure rates among nonsmoking patients ranged from 50% to 65%. Under the conditions of a 30% expected treatment failure rate among nonsmoking patients, 20% smoking prevalence, 60% increased risk of failure of first-line cancer treatment, and $100 000 mean added cost of treating a first-line cancer treatment failure, the additional incremental cost per 1000 total patients was estimated to be $2.1 million, reflecting an additional cost of $10 678 per smoking patient. Extrapolation of cost to 1.6 million patients with cancer diagnosed annually reflects a potential $3.4 billion in incremental cost.

**Conclusions and Relevance:**

The findings suggest that continued smoking among patients with cancer and the increase in attributable first-line cancer treatment failure is associated with significant incremental costs for subsequent cancer treatments. Additional work appears to be needed to identify optimal methods to mitigate these incremental costs.

## Key Points

**Question:**

What are the costs attributable to first-line cancer treatment failure associated with continued smoking in patients with cancer?

**Findings:**

In this economic evaluation, a model was developed to assess the cost associated with a broad spectrum of smoking prevalence, expected cure rates across different treatment and diagnostic conditions, risks from continued smoking, and cost of subsequent care. The attributable cost was $2.1 million per 1000 total patients, or $10 678 per smoking patient.

**Meaning:**

The findings suggest that continued smoking among patients with cancer is associated with substantial cancer treatment cost and justifies strategies to mitigate this incremental cost.

## Teaser

This economic evaluation models incremental costs of subsequent cancer treatment after failure of first-line cancer treatment among smoking vs nonsmoking patients with cancer.

## Introduction

The cost of cancer treatment is increasingly associated with unfavorable financial outcomes in cancer care,^1,2^ but whether increased costs reflect increased value is unknown. Principles for value-based care in oncology include patient-centered solutions, optimal care, and cost-containment strategies that do not limit patient access or innovation.^3^ Guidelines have been developed to define clinically meaningful outcomes in cancer care,^4^ but a recent analysis of drug approvals by the US Food and Drug Administration between 2014 and 2016 showed that many drugs did not meet survival goals.^5^ Whereas drug cost and efficacy have been the primary focus for considering value in cancer care, relatively little consideration has been given to other potentially modifiable factors that could affect cancer treatment costs, including health behaviors such as smoking.

The US 2014 Surgeon General’s report^6^ concluded that continued smoking among patients with cancer caused adverse outcomes including increased overall- and cancer-specific mortality, risk for second primary cancer, and associations with increased toxic effects from cancer treatment.^6^ Smoking cessation in the general population is known to improve health outcomes and create significant reductions in health expenditures.^6,7^ Whereas many studies found that smoking cessation after a cancer diagnosis can improve survival,^8,9,10,11,12,13^ to our knowledge, there have been no evaluations of the association between continued smoking among patients with cancer and the costs of cancer treatment. Understanding the financial effects of smoking among patients with cancer is needed to develop value-based approaches that could lead to improved cancer treatment outcomes. The objective of this study was to model the additional attributable first-line cancer treatment failures associated with continued smoking and to estimate the attributable incremental cost associated with the need to treat first-line cancer treatment failures attributed to continued smoking.

## Methods

### Model Design

For this economic evaluation, a model was developed in 2018 using data from the aforementioned 2014 US Surgeon General’s report.^6^ Smoking among patients with cancer and among cancer survivors increases risk for all-cause mortality, second primary cancer, and toxic effects from cancer treatment^6^; however, costs associated with noncancer comorbid disease management, end-of-life care, and complications associated with cancer treatment were not considered in this model. By estimating future costs of cancer treatment after failure of first-line cancer treatment and excluding other costs, cost estimates were expected to be conservative. Because this model used a publicly available resource, the Medical University of South Carolina, Charleston, deemed this study to be exempt from institutional review board approval. Reporting on this model complied with the Consolidated Health Economic Evaluation Reporting Standards (CHEERS) reporting guideline.

### Independent Model Parameters

Ideally, costs associated with continued smoking among patients with cancer could be applied to a variety of disease sites and conditions instead of being limited to homogeneous applications across cancer. The model could ideally be used to estimate effects across a spectrum of smoking prevalence, cure rates, risk of treatment failure attributed to smoking, and costs of subsequent treatment. Results could then be tailored to different clinical demographics and expected outcomes. To understand how continued smoking could affect costs associated with cancer treatment after failure of first-line treatment, the following measures were considered in the model: (1) expected probability of first-line cancer treatment failure rates in nonsmoking patients, (2) expected prevalence of smoking, (3) odds ratio (OR) of first-line cancer treatment failure for current smoking patients compared with nonsmoking patients, and (4) cost of cancer treatment after failure of first-line cancer treatment.

The expected cure rate, or effectiveness of first-line cancer treatment, varies considerably according to disease site, histologic features, stage, availability of effective therapeutic options, and other clinical variables.^14^ In general, higher cure rates are associated with lower stage of disease coupled with more effective therapeutic options. In this model, failures of first-line cancer treatment in nonsmoking patients were modeled across a wide spectrum, from 10% to 90%, to examine the incremental effects of failure rates associated with continued smoking under conditions that could be applied within a specific cure rate associated with any given cancer type and treatment.

Current smoking rates are highly dependent on geographic factors with variability in current smoking rates across cancer type.^6,15^ Lower current smoking rates have been reported among patients with breast or prostate cancer, whereas smoking rates of 50% or more have been reported in studies^6^ of patients with lung cancer. Studies^16,17,18^ have also shown that approximately 30% of patients with cancer who smoke may have misrepresented true tobacco use; thus, biochemically confirmed current smoking rates are expected to be higher than self-reported estimates. The model escalated the prevalence of smoking from 5% to 50% among patients with cancer.

The 2014 US Surgeon General’s report^6^ concluded that current smoking among patients with cancer caused an increase in cancer-specific mortality, with a median increased risk of 1.61 across cancer sites and treatments. In contrast, the median risk of cancer-related mortality among former smokers was 1.03. Risk of death due to cancer reflects failure of first-line cancer treatment and underestimates direct failure of first-line cancer treatment by discounting any health benefits beyond first-line cancer treatment. It is unclear what the increased risk of first-line treatment failure is according to disease site, histologic features, stage, treatment, and other clinical characteristics. For this analysis, given risks from the 2014 US Surgeon General’s report,^6^ the OR of failure of first-line cancer treatment among smoking patients compared with nonsmoking patients was modeled from 1.1 to 3.0.

### Cancer Treatment Failures Attributable to Smoking

For each combination of OR for treatment failure attributed to smoking, smoking prevalence, and expected baseline probability of failure of first-line cancer treatment in nonsmoking patients (FRns), the probability of failure of first-line cancer treatment in smoking patients (FRs) can be calculated by the following formula:

FRs = (FRns × OR)/(1 − FRns + OR × FRns)

In an overall cohort of smoking and nonsmoking patients, the expected number of treatment failures and cures are shown in Table 1. The number of first-line cancer treatment failures attributed to smoking is equal to the difference between the number of smoking patients who fail treatment and the number of smoking patients who would be expected to fail treatment if they were not smoking.

**Table 1.** Treatment Failure and Cure Calculations Among Smoking and Nonsmoking Patients With Cancer

Current Status	Treatment Failure^a^	Treatment Cure^b^	Total
Smoking	N × Ps × FRs	N × Ps × (1 − FRs)	N × Ps
Nonsmoking	N × (1 − Ps) × FRns	N × (1 − Ps) × (1 − FRns)	N × (1 − Ps)
Total	N × ([Ps × FRs] + [1 − Ps] × FRns)	N × (Ps × [1 − FRs] + [1 − Ps] × [1 − FRns])	N

Abbreviations: FRns, expected baseline probability of failure of first-line cancer treatment in nonsmoking patients; FRs, probability of failure of first-line cancer treatment in smoking patients; N, total cohort of smoking and non-smoking patients; Ps, smoking prevalence.

^a^ Expected number with failure of first-line cancer treatment.

^b^ Expected number with cure by first-line cancer treatment.

### Incremental Cost of Treatment Attributed to Smoking

The costs of cancer treatment are constantly changing and depend on multiple variables (eg, payer mix, billing procedures, clinical treatment pathways, and insurer agreements), but data have supported escalating costs for several years in part because of therapeutics used to manage failures of first-line cancer treatment.^1,19^ Rather than estimating costs according to treatment type (eg, surgery, radiotherapy, or systemic therapy) or individual cost structures within an institution or region, a defined incremental cost estimate per cancer treatment after failure of first-line cancer treatment was used. Costs per 1000 total patients and cost per smoking patient were estimated for individual costs of treating first-line cancer treatment failure to be between $10 000 and $250 000. Because this model was developed for fixed costs, these analyses can be adapted to changes in health care pricing and thus may be adaptable to future pricing in any time horizon.

### Statistical Analysis

The model was developed to estimate treatment failures in patients with cancer who smoke across a wide range of expected failure rates, risk from smoking, prevalence of smoking, and cost. This is a descriptive study with analyses performed using Microsoft Excel (Microsoft Corp).

## Results

The rate of first-line cancer treatment failure attributed to smoking increased with both the baseline failure rate in nonsmoking patients and risk associated with smoking, as shown in Table 2. When evaluated across a spectrum of expected treatment failure rates, treatment failures in smoking patients increased accordingly (eTable 1 in the Supplement). As failure rates increased in nonsmoking patients, failure rates in smoking patients increased because smoking prevalence and risk associated with smoking increased.

**Table 2.** Rate of Failure of First-line Cancer Treatment Among Smoking Patients vs Nonsmoking Patients

Odds Ratio^a^	Expected Baseline Probability of Failure of First-line Cancer Treatment Among Nonsmoking Patients						
	0.1	0.2	0.3	0.5	0.7	0.8	0.9
1.1	0.109	0.216	0.320	0.524	0.720	0.815	0.908
1.2	0.118	0.231	0.340	0.545	0.737	0.828	0.915
1.4	0.135	0.259	0.375	0.583	0.766	0.848	0.926
1.6	0.151	0.286	0.407	0.615	0.789	0.865	0.935
1.8	0.167	0.310	0.435	0.643	0.808	0.878	0.942
2.0	0.182	0.333	0.462	0.667	0.824	0.889	0.947
2.5	0.217	0.385	0.517	0.714	0.854	0.909	0.957
3.0	0.250	0.429	0.563	0.750	0.875	0.923	0.964

^a^ Odds ratio of first-line cancer treatment failure for current smoking patients compared with nonsmoking patients.

Treatment failures attributed to smoking were evaluated across a spectrum of expected failure rates among nonsmoking patients, risk associated with smoking, and smoking prevalence (eTable 2 in the Supplement). Instead of a linear or proportional increase in attributable failure across all baseline failure conditions, attributable treatment failures followed a curvilinear pattern, with higher effects seen under conditions with high cure rates (or low failure rates) among nonsmoking patients compared with conditions with low cure rates (or high failure rates). For example, in a cohort of patients with a 10% smoking prevalence and OR of 1.6, there were 5.1 attributable failures per 1000 total patients in cohorts with a 10% expected failure rate among nonsmoking patients (90% cure rate) compared with 3.5 attributable failures for cohorts with a 90% expected failure rate among nonsmoking patients (10% cure rate). The Figure shows the associations of escalating expected failure rates among nonsmoking patients and ORs with attributable failures in a cohort of patients with a smoking prevalence of 20%. In general, peak attributable failures occurred when the expected failure rate among nonsmoking patients ranged between 0.35 and 0.5, suggesting that the maximal association of smoking with first-line cancer treatment failure would occur under the conditions in which expected first-line cure rates ranged from 50% to 65%.

**Figure.**
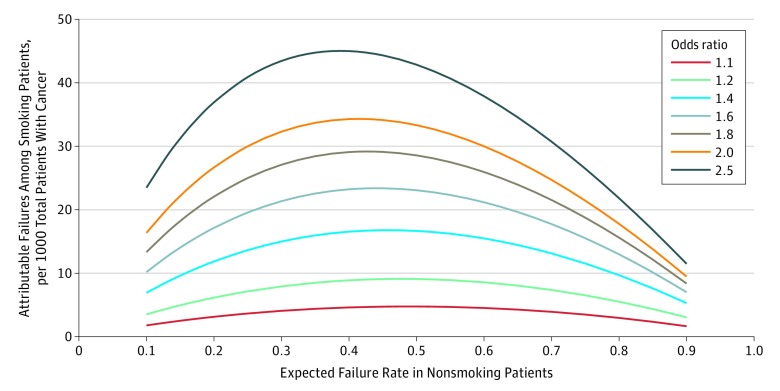
Attributable Failure per 1000 Total Patients Due to Continued Smoking

Table 3 provides costs associated with smoking per 1000 total patients based on a 30% failure rate in nonsmoking patients and 20% smoking prevalence. Because cancer treatment costs after first-line cancer treatment failure can vary substantially depending on disease site, failure pattern, and selection of subsequent therapy, model assumptions for cost of treating each attributable failure are shown for $10 000, $50 000, $100 000, and $250 000. For an OR of 1.6 and mean cost of treating first-line cancer treatment failure of $100 000, the cost per 1000 total patients was $2.1 million, reflecting a mean cost per smoking patient of $10 678 (Table 4). With attributable failure estimates, costs increased with ORs and maximal values were achieved for conditions in which expected cure rates were between 50% and 65%.

**Table 3.** Mean Cost Associated With First-line Cancer Treatment Failure Attributed to Smoking per 1000 Total Patients With a 30% Failure Rate of First-line Cancer Treatment Among Nonsmoking Patients and 20% Smoking Prevalence

Odds Ratio^a^	Mean Individual Cost per Treatment Failure, $			
	10 000	50 000	100 000	250 000
1.1	40 777	203 883	407 767	1 019 417
1.2	79 245	396 226	792 453	1 981 132
1.4	150 000	750 000	1 500 000	3 750 000
1.6	213 559	1 067 797	2 135 593	5 338 983
1.8	270 968	1 354 839	2 709 677	6 774 194
2.0	323 077	1 615 385	3 230 769	8 076 923
2.5	434 483	2 172 414	4 344 828	10 862 069
3.0	525 000	2 625 000	5 250 000	13 125 000

^a^ Odds ratio of first-line cancer treatment failure among smoking patients compared with nonsmoking patients.

**Table 4.** Cost per Smoking Patient Associated With $100 000 Mean Cost After Failure of First-line Cancer Treatment Based on 20% Smoking Prevalence

Odds Ratio^a^	Expected Probability of Failure of First-Line Cancer Treatment in Nonsmoking Patients, Cost, $								
	0.1	0.2	0.3	0.4	0.5	0.6	0.7	0.8	0.9
1.1	891	1569	2039	2308	2381	2264	1963	1481	826
1.2	1765	3077	3962	4444	4545	4286	3684	2759	1525
1.4	3462	5926	7500	8276	8333	7742	6563	4848	2647
1.6	5094	8571	10 678	11 613	11 538	10 588	8873	6486	3506
1.8	6667	11 034	13 548	14 545	14 286	12 973	10 769	7805	4186
2.0	8182	13 333	16 154	17 143	16 667	15 000	12 353	8889	4737
2.5	11 739	18 462	21 724	22 500	21 429	18 947	15 366	10 909	5745
3.0	15 000	22 857	26 250	26 667	25 000	21 818	17 500	12 308	6429

^a^ Odds ratio of first-line cancer treatment failure among smoking patients compared with nonsmoking patients.

## Discussion

Current smoking among patients with cancer was found to be associated with substantial increases in incremental cost for subsequent treatment after failure of first-line cancer treatment. Maximal effects were observed at expected cancer cure rates between 50% and 65% in nonsmoking patients. Extrapolating the outcomes from the 2014 US Surgeon General’s report^6^ of 20% smoking prevalence in 1.6 million patients annually with a 60% increased risk of failure attributed to smoking and $100 000 cost per cancer treatment failure, the results support a potential $3.4 billion incremental cost of treating cancer failures associated with continued smoking among patients with cancer in the United States each year. By providing estimates across smoking prevalence rates, risk of failure among smokers vs nonsmokers, and expected cure rates, this model may be applied to estimate outcomes across a breadth of potential cancer conditions and their associated costs. This model also estimates costs independent of potentially subjective assessments associated with determination of quality of life or confounding associated with cancer treatment toxic effects and non–cancer-related morbidity. Because comorbid conditions associated with smoking, such as heart disease and stroke, are known to increase annual medical expenditures among patients with cancer,^20^ estimates provided by this model are expected to be conservative.

The model was designed to be applicable to any defined cost of cancer treatment. Addressing treatment failures attributed to smoking will require a variety of approaches.^14^ Among patients with head and neck cancer who experience failure of curative surgery, radiotherapy, with or without chemotherapy, may be appropriate. For patients who experience failure of chemotherapy for lung cancer, second-line cytotoxic or biologic systemic therapy or immunotherapy may be indicated, with or without palliative conformal radiotherapy. For patients with esophageal cancer treated with definitive concurrent chemoradiotherapy, salvage esophagectomy or systemic therapy could be considered. Each disease condition and associated treatment costs are unique. Using this model and within a disease site and treatment modality, various costs can be assigned and combined with attributable treatment failures to estimate outcomes. This model can be tailored and applied to medical centers that specialize in specific cancer treatments or that have high- or low-smoking rates with a diversity of available disease sites or treatments.

The primary objective of this model was to assess whether smoking among patients with cancer is associated with significant additional treatment costs. Model results provide a foundation to develop strategies to reduce cancer treatment costs that are associated with smoking. In the context of cancer therapeutics, the incremental costs attributed to smoking can be significant. In 2011, a published estimate of the cost of cancer care in 2020 was $157 billion, based on adaptations to projected changes in population, but increased to $173 billion when a modest 2% increase in costs during the first and final year of life was considered.^21^ Although these projections continue to be listed online by the National Cancer Institute,^22^ recent analysis of the top 10 cancer drugs in 2015 in the United States supported an 8.8% annual increase in price,^23^ and a separate analysis supported a 10% annual increase in the launch price of anticancer drugs between 1995 and 2013.^24^ The increased estimates of anticancer drug prices have substantial cost implications. Simple calculations of cost differences amortized during 10 years realized a 22% overall increase at 2% annually compared with a 159% increase at 10% annually. Thus, avoiding any additional cancer treatment becomes increasingly important. There is no overall cost estimate for second-line cancer treatments across cancer sites and different failure patterns, but if a mean of $100 000 reflected true costs of treatment for each attributable failure, the cost burden associated with smoking would be substantial and comparable to the $3.5 billion estimated cost associated with pay for delay tactics.^25^ Any method to avoid additional medical costs through price reduction, reduced utilization through disease prevention, or better-defined clinical benefit may be justified. Interventions for clinicians that incorporated value-driven tools resulted in reduced costs for medical treatment.^26^ Estimates from this study placed outcomes at a high level and may justify methods that could prevent excess costs associated with continued smoking among patients with cancer.

The most pertinent question is whether attributable failures and associated costs can be mitigated with an existing evidence-based approach, such as smoking cessation. Several studies^8,9,10,11,12,13^ have shown that quitting smoking after a diagnosis of cancer is associated with improved overall survival. Most of these studies reported retrospective analyses of patients completing tobacco use assessments at diagnosis and follow-up. Few of these studies have evaluated whether enrolling patients in a smoking cessation program can affect overall or cancer-specific survival, but analysis^12^ of 224 patients with lung cancer who currently smoked and were enrolled in a telephone-based cessation program reported a 44% reduction in overall mortality associated with quitting smoking. Cessation of smoking after stereotactic radiotherapy was associated with a reduction in overall mortality of 52%.^10^ Cessation of smoking after treatment for small cell lung cancer was associated with a reduction in mortality of 41% compared with continued smoking.^9^ Although continued smoking after a diagnosis of breast cancer was associated with increased breast cancer mortality of 71%, cessation of smoking had no significant association.^27^ Although data support clinically meaningful survival and outcome benefits associated with smoking cessation among patients with cancer, to our knowledge, there are currently no large randomized clinical trials that have better defined this association. A method to realize the value of this treatment is widespread implementation of evidence-based smoking cessation programs in clinical practice. Survey results have reported that most oncologists do not provide smoking cessation support to patients with cancer.^28,29^ Most large cancer organizations and agencies are increasing awareness to develop dedicated smoking cessation resources for patients with cancer, including a recent effort by the National Cancer Institute to implement evidence-based smoking cessation approaches at National Cancer Institute designated cancer centers.^30^ Additional methods to address the clinical and cost effects of smoking during cancer treatment include determining whether there are existing cancer treatments that are not affected by smoking and the development of novel strategies to improve the therapeutic efficacy of cancer treatment among patients who smoke at the time of their cancer diagnosis.

### Limitations

There are several limitations to this study. No estimates of the cost of second-line cancer treatment exist that can be applied across cancer sites, treatment failure patterns, and second-line treatment options. We are unaware of any studies that have directly evaluated the costs of continued smoking associated with additional cancer treatment. However, the model facilitates selection of risk and cost estimates across a spectrum of costs for treating attributable failures. Whereas the cost of treating attributable failures was substantial, there are no data on the extent to which this cost burden can be reduced or eliminated. Smoking cessation could be the most evidence-based strategy, but it is unclear whether structured smoking cessation will partially or fully reverse attributable failures or what method of smoking cessation would be most effective. It is also unclear whether full abstinence is required, whether significant reductions in smoking are sufficient, and what length of time smoking reduction or abstinence before starting cancer treatment is required to manifest clinical benefits. Optimal strategies to facilitate smoking cessation during cancer care have not been defined, including whether more or less intensive interventions are superior when delivered in person, by telephone, or through hybrid approaches. Potential cost savings from smoking cessation may differ for different types of cancers and patient groups. To address these limitations, the model included a broad range of estimates across baseline failure rates, smoking prevalence, risk of treatment failure, and cost.

This model focused only on costs associated with addressing failure of first-line cancer treatment. Model estimates may be conservative because continued smoking may also be associated with increased risk of noncancer mortality, hospitalization, toxic effects of cancer treatment, and risk for second primary cancer,^6^ all of which would incur additional costs. However, the costs reflected in this analysis may not directly affect clinicians and may not be sufficient to motivate changes in clinical practice. If smoking cessation or more effective therapies can reverse the attributable failures and costs associated with smoking among patients with cancer, it is possible that the overall costs of long-term medical care may remain stable or increase. Interventions that reverse the effects of continued smoking should enable patients to live longer and without cancer recurrence. Increased patient longevity could accrue greater health care expenses and long-term medical conditions not associated with continued smoking. We believe that increased long-term medical costs associated with increased longevity because of reduced cancer recurrence is a good therapeutic objective.

## Conclusions

This model showed that continued smoking among patients with cancer was associated with significant incremental costs after failure of first-line cancer treatment. This model did not clarify the potential financial benefits of smoking cessation or alternative therapeutic cancer strategies. However, given the substantial attributable treatment failures and cost associated with continued smoking, development and implementation of effective mitigation strategies appear to be financially justified.

## Notes

Supplement. **eTable 1**. Expected Treatment Failures per 1000 Total Smoking Patients According to Failure Rate in Nonsmoking Patients (FRns) **eTable 2.** Attributable Failures per 1000 Total Smoking Patients According to Failure Rate in Nonsmoking Patients (FRns)
