# Supplementary material for: Attributable Failure of First-line Cancer Treatment and Incremental Costs Associated With Smoking by Patients With Cancer
Source: JAMA Netw Open. 2019 Apr 5;2(4):e191703. doi: 10.1001/jamanetworkopen.2019.1703 (PMC6450325; doi:10.1001/jamanetworkopen.2019.1703)
Supplement: Supplement. — eTable 1. Expected Treatment Failures per 1000 Total Smoking Patients According to Failure Rate in Nonsmoking Patients (FRns) eTable 2. Attributable Failures per 1000 Total Smoking Patients According to Failure Rate in Nonsmoking Patients (FRns) [file jamanetwopen-2-e191703-s001.pdf]

Supplementary Online Content

Warren GW, Cartmell KB, Garrett-Mayer E, Salloum RG, Cummings KM. Attributable failure of first-line cancer treatment and incremental costs associated with smoking by patients with cancer. *JAMA Netw Open*. 2019;2(4):e191703. doi:10.1001/jamanetworkopen.2019.1703

eTable 1. Expected Treatment Failures per 1000 Total Smoking Patients According to Failure Rate in Nonsmoking Patients (FRns)

eTable 2. Attributable Failures per 1000 Total Smoking Patients According To Failure Rate In Nonsmoking Patients (FRns)

This supplementary material has been provided by the authors to give readers additional information about their work.

© 2019 Warren GW et al. *JAMA Network Open*.

eTable 1. Expected Treatment Failures per 1000 Total Smoking Patients According to Failure Rate in Nonsmoking Patients (FRns). Abbreviations: FRns = expected baseline probability of failure rate of primary cancer treatment in non-smoking patients, TFs = expected number of smoking patients who fail primary cancer treatment, Ps = prevalence of smoking, ORs = odds ratio of primary cancer treatment failure for current smoking patients as compared with non-smoking patients.

Expected treatment failures in smoking patients (TFs) per 1000 total cancer patients when FRns is very low (10%)

ORs	Ps=0.05	Ps=0.1	Ps=0.15	Ps=0.2	Ps=0.3	Ps=0.4	Ps=0.5
**1.1**	5.4	10.9	16.3	21.8	32.7	43.6	54.5
**1.2**	5.9	11.8	17.6	23.5	35.3	47.1	58.8
**1.4**	6.7	13.5	20.2	26.9	40.4	53.8	67.3
**1.6**	7.5	15.1	22.6	30.2	45.3	60.4	75.5
**1.8**	8.3	16.7	25.0	33.3	50.0	66.7	83.3
**2.0**	9.1	18.2	27.3	36.4	54.5	72.7	90.9
**2.5**	10.9	21.7	32.6	43.5	65.2	87.0	108.7
**3.0**	12.5	25.0	37.5	50.0	75.0	100.0	125.0

Expected treatment failures in smoking patients (TFs) per 1000 total cancer patients when FRns is low (30%)

ORs	Ps=0.05	Ps=0.1	Ps=0.15	Ps=0.2	Ps=0.3	Ps=0.4	Ps=0.5
**1.1**	16.0	32.0	48.1	64.1	96.1	128.2	160.2
**1.2**	17.0	34.0	50.9	67.9	101.9	135.8	169.8
**1.4**	18.8	37.5	56.3	75.0	112.5	150.0	187.5
**1.6**	20.3	40.7	61.0	81.4	122.0	162.7	203.4
**1.8**	21.8	43.5	65.3	87.1	130.6	174.2	217.7
**2.0**	23.1	46.2	69.2	92.3	138.5	184.6	230.8
**2.5**	25.9	51.7	77.6	103.4	155.2	206.9	258.6
**3.0**	28.1	56.3	84.4	112.5	168.8	225.0	281.3

© 2019 Warren GW et al. *JAMA Network Open*.

Expected treatment failures in smoking patients (TFs) per 1000 total cancer patients when FRns is 50%

ORs	Ps=0.05	Ps=0.1	Ps=0.15	Ps=0.2	Ps=0.3	Ps=0.4	Ps=0.5
**1.1**	26.2	52.4	78.6	104.8	157.1	209.5	261.9
**1.2**	27.3	54.5	81.8	109.1	163.6	218.2	272.7
**1.4**	29.2	58.3	87.5	116.7	175.0	233.3	291.7
**1.6**	30.8	61.5	92.3	123.1	184.6	246.2	307.7
**1.8**	32.1	64.3	96.4	128.6	192.9	257.1	321.4
**2.0**	33.3	66.7	100.0	133.3	200.0	266.7	333.3
**2.5**	35.7	71.4	107.1	142.9	214.3	285.7	357.1
**3.0**	37.5	75.0	112.5	150.0	225.0	300.0	375.0

Expected treatment failures in smoking patients (TFs) per 1000 total cancer patients when FRns is high (70%)

ORs	Ps=0.05	Ps=0.1	Ps=0.15	Ps=0.2	Ps=0.3	Ps=0.4	Ps=0.5
**1.1**	36.0	72.0	107.9	143.9	215.9	287.9	359.8
**1.2**	36.8	73.7	110.5	147.4	221.1	294.7	368.4
**1.4**	38.3	76.6	114.8	153.1	229.7	306.3	382.8
**1.6**	39.4	78.9	118.3	157.7	236.6	315.5	394.4
**1.8**	40.4	80.8	121.2	161.5	242.3	323.1	403.8
**2.0**	41.2	82.4	123.5	164.7	247.1	329.4	411.8
**2.5**	42.7	85.4	128.0	170.7	256.1	341.5	426.8
**3.0**	43.8	87.5	131.3	175.0	262.5	350.0	437.5

Expected treatment failures in smoking patients (TFs) per 1000 total cancer patients when FRns is very high (90%)

ORs	Ps=0.05	Ps=0.1	Ps=0.15	Ps=0.2	Ps=0.3	Ps=0.4	Ps=0.5
**1.1**	45.4	90.8	136.2	181.7	272.5	363.3	454.1
**1.2**	45.8	91.5	137.3	183.1	274.6	366.1	457.6
**1.4**	46.3	92.6	139.0	185.3	277.9	370.6	463.2
**1.6**	46.8	93.5	140.3	187.0	280.5	374.0	467.5
**1.8**	47.1	94.2	141.3	188.4	282.6	376.7	470.9
**2.0**	47.4	94.7	142.1	189.5	284.2	378.9	473.7
**2.5**	47.9	95.7	143.6	191.5	287.2	383.0	478.7
**3.0**	48.2	96.4	144.6	192.9	289.3	385.7	482.1

© 2019 Warren GW et al. *JAMA Network Open*.

eTable 2: Attributable Failures per 1000 Total Smoking Patients According to Failure Rate in Nonsmoking Patients (FRns). Abbreviations: AFs = attributable failures of primary cancer treatment due to smoking, FRns = expected baseline probability of failure rate of primary cancer treatment in non-smoking patients, Ps = prevalence of smoking, ORs = odds ratio of primary cancer treatment failure for current smoking patients as compared with non-smoking patients.

Attributable failures in smoking patients (AFs) per 1000 total cancer patients when FRns is very low (10%)

ORs	Ps=0.05	Ps=0.1	Ps=0.15	Ps=0.2	Ps=0.3	Ps=0.4	Ps=0.5
**1.1**	0.4	0.9	1.3	1.8	2.7	3.6	4.5
**1.2**	0.9	1.8	2.6	3.5	5.3	7.1	8.8
**1.4**	1.7	3.5	5.2	6.9	10.4	13.8	17.3
**1.6**	2.5	5.1	7.6	10.2	15.3	20.4	25.5
**1.8**	3.3	6.7	10.0	13.3	20.0	26.7	33.3
**2.0**	4.1	8.2	12.3	16.4	24.5	32.7	40.9
**2.5**	5.9	11.7	17.6	23.5	35.2	47.0	58.7
**3.0**	7.5	15.0	22.5	30.0	45.0	60.0	75.0

Attributable failures in smoking patients (AFs) per 1000 total cancer patients when FRns is low (30%)

ORs	Ps=0.05	Ps=0.1	Ps=0.15	Ps=0.2	Ps=0.3	Ps=0.4	Ps=0.5
**1.1**	1.0	2.0	3.1	4.1	6.1	8.2	10.2
**1.2**	2.0	4.0	5.9	7.9	11.9	15.8	19.8
**1.4**	3.8	7.5	11.3	15.0	22.5	30.0	37.5
**1.6**	5.3	10.7	16.0	21.4	32.0	42.7	53.4
**1.8**	6.8	13.5	20.3	27.1	40.6	54.2	67.7
**2.0**	8.1	16.2	24.2	32.3	48.5	64.6	80.8
**2.5**	10.9	21.7	32.6	43.4	65.2	86.9	108.6
**3.0**	13.1	26.3	39.4	52.5	78.8	105.0	131.3

Attributable failures in smoking patients (AFs) per 1000 total cancer patients when FRns is 50%

ORs	Ps=0.05	Ps=0.1	Ps=0.15	Ps=0.2	Ps=0.3	Ps=0.4	Ps=0.5
**1.1**	1.2	2.4	3.6	4.8	7.1	9.5	11.9
**1.2**	2.3	4.5	6.8	9.1	13.6	18.2	22.7
**1.4**	4.2	8.3	12.5	16.7	25.0	33.3	41.7
**1.6**	5.8	11.5	17.3	23.1	34.6	46.2	57.7
**1.8**	7.1	14.3	21.4	28.6	42.9	57.1	71.4
**2.0**	8.3	16.7	25.0	33.3	50.0	66.7	83.3
**2.5**	10.7	21.4	32.1	42.9	64.3	85.7	107.1
**3.0**	12.5	25.0	37.5	50.0	75.0	100.0	125.0

© 2019 Warren GW et al. *JAMA Network Open*.

Attributable failures in smoking patients (AFs) per 1000 total cancer patients when FRns is high (70%)

ORs	Ps=0.05	Ps=0.1	Ps=0.15	Ps=0.2	Ps=0.3	Ps=0.4	Ps=0.5
**1.1**	1.0	2.0	2.9	3.9	5.9	7.9	9.8
**1.2**	1.8	3.7	5.5	7.4	11.1	14.7	18.4
**1.4**	3.3	6.6	9.8	13.1	19.7	26.3	32.8
**1.6**	4.4	8.9	13.3	17.7	26.6	35.5	44.4
**1.8**	5.4	10.8	16.2	21.5	32.3	43.1	53.8
**2.0**	6.2	12.4	18.5	24.7	37.1	49.4	61.8
**2.5**	7.7	15.4	23.0	30.7	46.1	61.5	76.8
**3.0**	8.8	17.5	26.3	35.0	52.5	70.0	87.5

Attributable failures in smoking patients (AFs) per 1000 total cancer patients when FRns is very high (90%)

ORs	Ps=0.05	Ps=0.1	Ps=0.15	Ps=0.2	Ps=0.3	Ps=0.4	Ps=0.5
**1.1**	0.4	0.8	1.2	1.7	2.5	3.3	4.1
**1.2**	0.8	1.5	2.3	3.1	4.6	6.1	7.6
**1.4**	1.3	2.6	4.0	5.3	7.9	10.6	13.2
**1.6**	1.8	3.5	5.3	7.0	10.5	14.0	17.5
**1.8**	2.1	4.2	6.3	8.4	12.6	16.7	20.9
**2.0**	2.4	4.7	7.1	9.5	14.2	18.9	23.7
**2.5**	2.9	5.7	8.6	11.5	17.2	23.0	28.7
**3.0**	3.2	6.4	9.6	12.9	19.3	25.7	32.1

© 2019 Warren GW et al. *JAMA Network Open*.
